# IRVING S. WRIGHT AWARD, VINCENT CRISTOFALO AWARD, AND TERRIE FOX WETLE AWARD PRESENTATIONS AND LECTURES

**DOI:** 10.1093/geroni/igad104.1356

**Published:** 2023-12-21

**Authors:** Steven Austad

## Abstract

The Irving S. Wright Award of Distinction Lecture will feature an address by the 2023 recipient Rafael De Cabo, BS, FGSA of National Institute on Aging, National Institutes of Health. The Vincent Cristofalo Rising Star Award in Aging Research lecture will feature an address by the 2023 recipient Ming Xu, PhD of UConn Health. The Terrie Fox Wetle Award lecture will feature an address by the 2023 recipient Claire Ankunda, MD, MPH. MSc of Icahn School of Medicine at Mount Sinai. These awards are given by the American Federation for Aging Research, Inc.

